# *Meta*-Topolin-induced mass shoot multiplication and biosynthesis of valuable secondary metabolites in *Stevia rebaudiana* Bertoni bioreactor culture

**DOI:** 10.1038/s41598-023-42619-8

**Published:** 2023-09-19

**Authors:** Agata Ptak, Agnieszka Szewczyk, Magdalena Simlat, Alicja Błażejczak, Marzena Warchoł

**Affiliations:** 1 Department of Plant Breeding, Physiology and Seed Science, University of Agriculture in Krakow, Łobzowska 24, 31-140 Krakow, Poland; 2 Department of Pharmaceutical Botany, Faculty of Pharmacy, Jagiellonian University Medical College, Medyczna 9, 30-688 Krakow, Poland; 3 The Franciszek Górski Institute of Plant Physiology, Polish Academy of Sciences, Niezapominajek 21, 30-239, Kraków, Poland

**Keywords:** Plant biotechnology, Plant hormones, Secondary metabolism

## Abstract

*Stevia rebaudiana* Bertoni possesses various medicinal and food industrial applications. This study is the first to explore the effect of the cytokinins *meta*-Topolin (*m*T; 6-(3-hydroxybenzylamino) purine), zeatin, kinetin, and BAP (6-benzylaminopurine) at concentrations of 0 (control), 5, 10, and 15 µM on shoot multiplication, as well as stevioside, rebaudioside A, phenolic acid, and flavonoid content in bioreactor cultures. The highest number of shoots (23.4 per explant) was obtained in the medium containing 5 μM of* m*T. However, 15 μM of *m*T was superior for fresh biomass production and dry biomass accumulation. Reversed-phase (RP)-HPLC analysis showed a beneficial effect of 5 μM *m*T on stevioside (11.43 mg/g dry weight [DW]) and rebaudioside A (10.74 mg/g DW) biosynthesis. In all conditions, the ratio of rebaudioside A/stevioside ranged from 0.75 to 1.12. The phenolic acids chlorogenic, neochlorogenic, isochlorogenic A, and rosmarinic were confirmed in the stevia extracts, as were the flavonoids isoquercetin, and quercitrin. The highest accumulations of chlorogenic and neochlorogenic acids and flavonoids were observed in shoot tissues derived from 5 µM *m*T, whereas 5 µM of BAP stimulated biosynthesis of chlorogenic, isochlorogenic A, and rosmarinic acids. This is the first report on the use of *m*T-cytokinin showing high potential in stevia cultures.

## Introduction

Stevia (*Stevia rebaudiana* Bertoni, Asteraceae) is a perennial herbaceous medicinal plant originating from Paraguay containing compounds that are about 300 times sweeter than sucrose^1^. Its sweetness is due to the presence of *ent*-kaurene type diterpene steviol glycosides (SG), such as stevioside and rebaudioside A^2^. As a noncaloric, natural sweetener, SG is used as a sugar substitute in the food industry (approved for consumption by the US Food and Drug Administration in 2008 and by the European Union in 2011)^3^. Other bioactive compounds isolated from stevia include polyphenols, flavonoids, tannins and alkaloids^4^. Stevia leaves are also a good source of vitamins, essential amino acids, minerals and fatty acids^5^. Stevia has become a very important medicinal plant because of its excellent therapeutic effects through anti-hyperglycaemic, anti-inflammatory, anti-tumour, antioxidant, anti-hypertensive, anti-obesity, anti-caries and anti-cancer properties^6^.

Stevia is commercially cultivated in many countries, including Argentina, Brazil, Columbia, Paraguay, China, Japan, Malaysia, South Korea, Vietnam, Israel, Australia, Kenya and the United States^7^. The plants are propagated from seeds and vegetatively from stem cuttings. However, the poor viability of stevia seeds and length of time required for seedling development do not allow for the commercialisation of this method. Additionally, propagation using the seeds can also cause great variability in stevioside levels^8^. Propagation by stem cutting is limited because of the minimal number of plants that can be obtained simultaneously from a single plant^9^. An alternative to conventional propagation methods may be in vitro micropropagation of stevia. Indeed, leaf fragments, shoot tips, nodal segments, axillary buds, anthers and flowers have all been used as explants for the initiation of in vitro stevia cultures^10–13^.

Several micropropagation protocols have been established in which organogenesis is a more frequently used morphogenetic pathway for stevia plant propagation than somatic embryogenesis^14^. To develop an efficient method of micropropagation and the possibility of secondary metabolite production in in vitro conditions, different types of cultures have been conducted, including callus, suspension, shoot and root (adventitious and transformed)^15–17^. However, there is no fully developed micropropagation protocol that would allow for the production of a large number of high-quality plants in a short time that would be economically viable and could be used for industrial applications.

Similarly, many studies have been conducted on the biosynthesis of stevioside and rebaudioside A in in vitro cultures of stevia, but the industrial production of SG using biotechnology has still not been implemented because of the poor performance of the target compounds. Detailed information on this subject can be found in the research of Miladinova-Georgieva et al.^14^.

Some studies have also determined the total content of phenolic compounds in the stevia plant material obtained in vitro, usually as stress markers^18^. However, the research on the biosynthesis of phenolic acids in stevia cultures has been fragmentary, at best. Only Fu et al.^19^ have demonstrated the possibility of biosynthesis of chlorogenic acids and their derivatives in stevia hairy root culture. It is known that phenolic acids possess excellent hydrophilic antioxidant activity and other therapeutic properties^20^. Flavonoids are another important group of phenolic compounds isolated from stevia leaves with regard to, for example, antioxidant and anti-proliferative activities^21^. The available literature lacks information regarding determining the content of individual flavonoids in in vitro cultures of stevia. Only Blinstrubienė et al.^22^ and Idrees et al.^23^ have determined the total flavonoid content in the callus and roots of stevia. For this reason, studies of individual phenolic acid and flavonoid production from in vitro stevia cultures needs to be expanded.

In studies on the biosynthesis of secondary metabolites in stevia cultures, elicitors have usually been tested^24^. Elicitation is a technique involving the addition of abiotic or biotic elicitors to the medium, which triggers a stress response, thus increasing the production of secondary metabolites. In in vitro cultures of stevia, elicitors that have been used include methyl jasmonate, spermidine, salicylic acid, paclobutrazol, gibberellic acids and different combinations of auxins and cytokinins^25–27^. Among the cytokinins, kinetin and BAP are usually applied^27^. A natural cytokinin, *m*T has been recognised as an excellent elicitor of secondary metabolite biosynthesis, e.g. galanthamine, phenolic acids and flavonoids, but it has never been tested in stevia cultures^28–30^. However, a beneficial effect of *m*T has been demonstrated for improved multiplication rate and in vitro plant quality^28,31^.

In addition, *m*T causes better rooting and plant acclimatisation^32^. Moreover,* m*T is considered to be a cytokinin that does not cause hyperhydricity and could therefore be a substitute for BAP, which can cause genetic changes and induce physiological disorders and hyperhydricity^32^. Apart from the appropriate selection of cytokinins, another method of reducing hyperhydricity is the use of bioreactor cultures carried out in a temporary immersion system^28^.

The aim of the present work was to, for the first time, optimise the following: (1) mass shoot multiplication, (2) the production of SG (stevioside and rebaudioside A) and (3) the biosynthesis of phenolic compounds (phenolic acids and flavonoids) in the stevia temporary immersion bioreactor Rita^®^ through the use of treatment of different cytokinins. In addition, this is the first report on the use of *m*T in micropropagation of stevia and secondary metabolite production. To the best of our knowledge, there is no detailed information on individual phenolic acid and flavonoid content in stevia in vitro shoots. The proposed research will improve the multiplication process and phytochemical traits of stevia for industrial applications.

## Materials and methods

### Plant materials

Seeds of *Stevia rebaudiana* Bertoni obtained from W. Legutko Breeding and Seed Company, Poland, were surface sterilised in 70% ethanol for 30 s and then in 20% Domestos (containing 5% chloride, Unilever, Poland) for 15 min. After sterilisation, the seeds were rinsed three times with sterile water and plated on a Murashige and Skoog (MS)^33^ medium supplemented with 6 g/L agar, at pH 5.8. The germination was conducted in a growth chamber at 25 ± 1 °C under white fluorescent light with a 16 h photoperiod (Tungsram lamp, 40 WF, 90 μmol m^−2^ s^−1^). The obtained seedlings were transferred for four weeks of growth to the same medium as for germination. Next, nodal segments (1.5 ± 0.5 cm in length) were isolated from stevia plantlets and used as the initial materials for the experiment. The authors confirm that all methods used were performed in accordance with the relevant guidelines and legislation.

### Cytokinin treatment and in vitro culture conditions

The nodal explants with axillary buds and leaves were cultured in a temporary immersion bioreactor Rita^®^ (Vitropic, France). The immersion frequency was 5 min every 2 h, as described by Ptak et al.^28^. The cultures were kept at 25 ± 2 °C under white fluorescent light with a 16 h photoperiod (Tungsram lamp, 40 WF, 90 µmol m^−2^ s^−1^). For inoculation, 15 nodal explants (total fresh weight [FW] of shoots: about 3 g) were transferred to bioreactor vessels containing 200 mL MS liquid medium without growth regulators (control) and enriched with the cytokinins: *m*T, zeatin, kinetin and BAP all at the same concentrations: 5, 10 or 15 µM. The experiment was repeated three times. For each combination, 45 explants were cultured; for the total experiment, 585 explants were used.

After four weeks of culture, the number of shoots developed from one nodal explant that was counted, and FW biomass increments per one bioreactor vessel were calculated.

To determine the accumulation of DW, the shoots were freeze-dried for 72 h and then weighed. The DW content in the plant tissue was calculated according to the following formula: DW [%] = DW_L_ × 100/FW_F_, where DW_L_ is the dry weight after lyophilisation and FW_F_ is the final fresh weight. Lyophilised and powdered samples were stored at − 80 °C for further analysis.

### Analyses of steviol glycoside

Lyophilised and powdered samples (about 250 mg) were extracted with 2 mL of methanol for 1 h at 30 °C. The supernatant was filtered through 0.22 μm filter (Millex^®^GP, Millipore, Merck, Darmstadt, Germany). RP-HPLC analysis was carried out on a Merck–Hitachi liquid chromatograph (LaChrom Elite) equipped with a DAD detector L-2455 and a Purospher RP-18e column as previously described by Simlat et al.^8^. Detection was performed at λ = 210 nm, and the UV spectra of all samples were scanned between 190 and 360 nm. Quantification was defined by measuring the peak area with reference to the standard curve derived from five concentrations (from 0.0625 to 1 mg ml^−1^) of authentic reference SG: stevioside and rebaudioside A (acquired from Sigma-Aldrich Co., Germany). The analysis was repeated three times.

### Analyses of phenolic acids and flavonoids

Lyophilised and powdered samples (about 250 mg) were extracted with 2 mL of methanol using sonication (10 min at 25 ± 2 °C) and centrifuged for 10 min at 15,000 rpm. The supernatant was filtered through 0.22 μm filter (Millex^®^GP, Millipore, Merck, Darmstadt, Germany). RP-HPLC analyses was carried out according to Simlat et al.^34^ using a Merck–Hitachi liquid chromatograph (LaChrom Elite, Hitachi, Tokyo, Japan) equipped with a DAD detector L-2455 and Purospher^®^RP-18e (250 × 4 mm/5 mm) column (Merck, Darmstadt, Germany). Quantification was carried out at λ = 254 nm for phenolic acids and λ = 370 nm for flavonoids. Identification was performed through a comparison of the retention times of the peaks with authentic reference compounds and co-chromatography with standards. Quantification consisted of measurement of the peak area with reference to the standard curve derived from five concentrations (0.03125–0.5 mg ml^−1^). Commercially available standards of the phenolic acids: chlorogenic, neochlorogenic (Sigma–Aldrich, St Louis, MO, USA), isochlorogenic A (ChromaDex, Irvine, CA, USA), and rosmarinic (Sigma–Aldrich, St Louis, MO, USA), as well as the flavonoids isoquercetin, and quercitrin (Sigma–Aldrich, St Louis, MO, USA), were used to generate the calibration curves. The analysis was repeated three times.

### Data analysis

Statistical analysis of the data was performed using analysis of variance (ANOVA). Differences between the means were determined using Duncan’s multiple range test at *P* < 0.05. The values are expressed as the means ± standard deviation (SD).

## Results

### Influence of cytokinins on shoot appearance, multiplications and biomass increments

**Figure 1:**
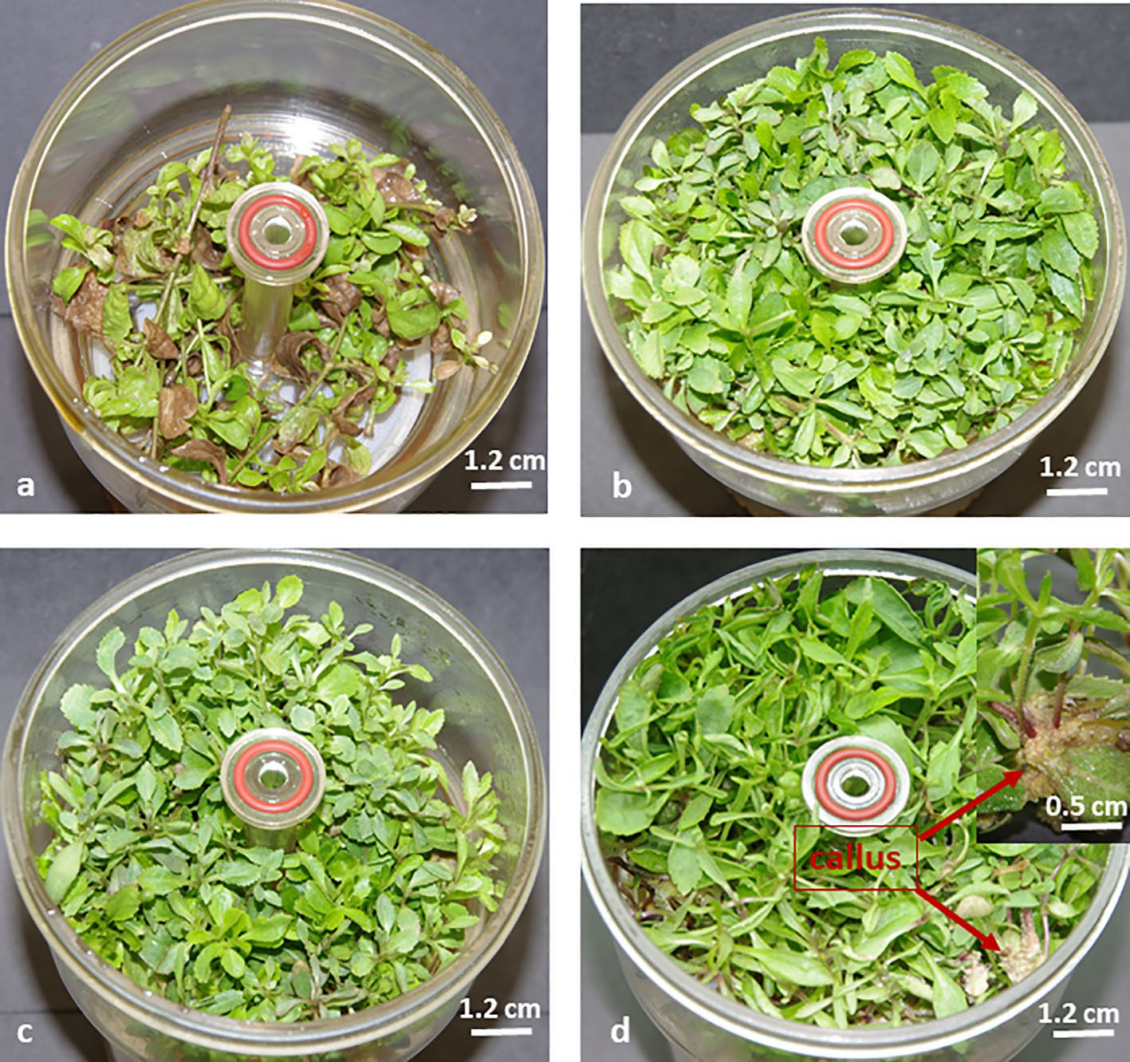
Morphological appearance of *Stevia rebaudiana* microshoots grown on media supplemented with different concentrations of *meta*-Topolin (**a**) control (0), (**b**) 5 µM, (**c**) 10 µM, (**d**) 15 µM.

The exogenous cytokinin had a positive effect on *Stevia rebaudiana* in vitro shoot appearance and growth in bioreactor Rita^®^. In general, the stevia cultivated for four weeks on the media with the addition of cytokinin (irrespective of type and concentration) were characterised by a green colour of leaves, while the leaves and shoots derivate from the control medium (without cytokinin) were necrotic and tended to turn brown and dying (Fig. 1). No stevia shoots showed signs of vitrification, including those obtained on media with the addition of cytokinins at the highest concentration (Fig. 1d). Meanwhile, we observed callus formation based on stevia shoots growing on the medium containing 15 µM of tested cytokinin (Fig. 1d).

Table 1 shows that the cytokinin type and its concentration significantly affected the number of shoots/explant after four weeks of cultivation. The highest number of shoots (23.43 shoots per explant, 351.50 shoots per bioreactor) was observed under the influence of 5 µM of *m*T. Slightly smaller amounts of shoots were formed when 10 µM and 15 µM *m*T were used (20.57 and 21.20 shoots per explant, 308.50 and 318 shoots per bioreactor, respectively), though the fewest shoots were noted on the medium without cytokinin (10.10 shoots per explant, 151.50 shoots per bioreactor) and when the medium was enriched with kinetin (on average: 11.48 shoots per explant, 172.17 shoots per bioreactor).

**Table 1:** Effect of different cytokinins on in vitro *Stevia rebaudiana* shoot multiplication.

Cytokinin [µM]	Mean number of shoots/explant	Total number of shoots/bioreactor	FW biomass increments/bioreactor [g]	DW accumulation in shoots/bioreactor [%]
Control (0)	10.10 ± 6.25h	151.50 ± 9.38h	22.47 ± 0.36f	1.80 ± 0.03f
*m*T 5	23.43 ± 6.99a	351.50 ± 10.49a	57.38 ± 0.20b	4.59 ± 0.01b
*m*T 10	20.57 ± 1.33b	308.50 ± 2.00b	59.42 ± 0.90b	4.75 ± 0.07b
*m*T 15	21.20 ± 3.47b	318.00 ± 5.20b	72.27 ± 0.10a	5.78 ± 0.01a
Zeatin 5	12.47 ± 4.85fg	187.00 ± 7.27fg	44.60 ± 1.83d	3.57 ± 0.15d
Zeatin 10	13.73 ± 1.33ef	206.00 ± 2.00ef	45.47 ± 0.32d	3.64 ± 0.02d
Zeatin 15	14.77 ± 6.97de	221.00 ± 10.45de	49.01 ± 2.12c	3.92 ± 0.17c
Kinetin 5	13.13 ± 6.37ef	197.00 ± 9.55ef	39.40 ± 0.10e	3.15 ± 0.08e
Kinetin 10	10.33 ± 7.27h	155.00 ± 10.91h	43.70 ± 0.50d	3.50 ± 0.04d
Kinetin 15	10.97 ± 11.15gh	164.50 ± 16.72gh	46.32 ± 1.17cd	3.71 ± 0.09cd
BAP 5	16.33 ± 3.53cd	245.00 ± 5.30cd	40.20 ± 0.72e	3.22 ± 0.06e
BAP 10	16.54 ± 1.20c	248.00 ± 1.80c	44.55 ± 0.46d	3.56 ± 0.04d
BAP 15	17.20 ± 2.61c	258.00 ± 3.91c	46.28 ± 1.33cd	3.70 ± 0.11cd

Different letters indicate a significance difference at *P* < 0.05 according to ANOVA and Duncan’s test; the values are expressed as the means of three replicates (n = 3) ± standard deviation (SD).

*m*T *meta*-Topolin, *BAP* 6-benzylaminopurine, *FW* fresh weight, *DW* dry weight.

The results from the present study show that the highest increase in FW of stevia shoot biomass was recorded when the medium containing 15 µM of *m*T was used (72.27 g/bioreactor). These increments were 3.22 times greater compared with the control, where the lowest value was noted (22.47 g/bioreactor). Among the tested cytokinins, regardless of the concentration used, the strongest effect was observed in the case of *m*T (FW increments: 63.03 g), followed by zeatin (FW increments: 43.36 g), BAP (FW increments: 43.68 g) and kinetin (FW increments: 43.14 g) (Table 1). The same trends were observed in the accumulation of DW of shoots; that is, the highest accumulation of DW was observed for shoots propagated on a medium with the addition of *m*T (5.04%); the concentration of 15 µM *m*T was the most effective (5.78); for comparison, in the control group, it was 1.80% (Table 1).

### Influence of cytokinins on the accumulation of steviol glycosides

RP-HPLC analyses of methanol extracts from the shoot biomass of stevia bioreactor cultures confirmed the presence of SG: stevioside and rebaudioside A under all tested conditions (Table 2, Supplementary Fig. S1 online). However, Table 2 shows that the amounts of the measured compounds depended on the type and concentration of cytokinin treatment applied to the in vitro shoots. *M*T at a concentration of 5 µM, was the most favourable for stevioside production in the stevia culture (11.43 mg/g DW). Slightly lower amounts of stevioside were determined in the shoots growing in the medium with the addition of 10 µM of *m*T and in the control medium (8.78 and 8.64 mg/g DW, respectively). The lowest content of stevioside was recorded in shoots cultivated in the medium enriched with 15 µM of kinetin (3.71 mg/g DW). It is also worth emphasising that the accumulation of stevioside in bioreactor biomass, achieved with a medium containing 5 µM of *m*T (DW content in biomass from bioreactor: 4.59%, 351.50 shoots), was 3.37 times greater than that obtained with a medium without cytokinin (DW content in biomass from bioreactor: 1.80%, 151.50 shoots) (Tables 1, 2).

**Table 2:** Effect of different cytokinins on stevioside and rebaudioside A biosynthesis in in vitro culture of *Stevia rebaudiana*.

Cytokinins [µM]	Stevioside [mg/g DW]	Rebaudioside A [mg/g DW]	Stevioside/bioreactor [mg DW]	Rebaudioside A/bioreactor [mg DW]	Rebaudioside A/stevioside ratio
Control 0	8.64 ± 0.43b	6.56 ± 0.14c	15.55 ± 1.01ef	11.80 ± 0.44f	0.76 ± 0.02e
*m*T 5	11.43 ± 0.14a	10.74 ± 0.11a	52.46 ± 0.59a	49.31 ± 0.34a	0.94 ± 0.01bcd
*m*T 10	8.78 ± 0.20b	7.73 ± 0.11 b	41.75 ± 1.56b	36.75 ± 0.79b	0.88 ± 0.02b-e
*m*T 15	4.42 ± 0.05de	4.59 ± 0.08efg	25.58 ± 0.27c	26.54 ± 0.47c	1.04 ± 0.02ab
Zeatin 5	4.42 ± 0.13de	4.36 ± 0.18efg	15.82 ± 1.09ef	15.61 ± 1.23efg	0.99 ± 0.03abc
Zeatin 10	3.64 ± 0.04e	3.58 ± 0.21g	13.22 ± 0.19f	13.02 ± 0.86fg	0.98 ± 0.05abc
Zeatin 15	4.77 ± 0.05de	5.36 ± 0.08de	18.68 ± 0.63def	20.98 ± 0.67d	1.12 ± 0,01a
Kinetin 5	6.89 ± 0.49c	6.05 ± 0.4cd	21.71 ± 1.36cde	19.06 ± 0.83de	0.88 ± 0.03cde
Kinetin 10	5.78 ± 0.58cd	4.61 ± 0.35efg	20.16 ± 1.89cde	16.08 ± 1.23efg	0.80 ± 0.07de
Kinetin 15	3.71 ± 1.30e	4.06 ± 0.96fg	13.79 ± 4.38f	15.06 ± 3.22efg	1.09 ± 0.04a
BAP 5	6.93 ± 0.06c	5.20 ± 0.07def	22.25 ± 0.36cd	16.70 ± 0.55ef	0.75 ± 0.02e
BAP 10	5.09 ± 0.90de	4.19 ± 0.66efg	18.12 ± 2.98def	14.94 ± 2.16efg	0.82 ± 0.11cde
BAP 15	4.29 ± 0.62de	3.61 ± 0.42g	15.67 ± 2.51ef	13.22 ± 1.76fg	0.84 ± 0.07cde

Different letters indicate a significance difference at *P* < 0.05 according to ANOVA and Duncan’s test; the values are expressed as the means of three replicates (n = 3) ± standard deviation (SD).

*m*T *meta*-Topolin, *BAP* 6-benzylaminopurine, *DW* dry weight.

The treatment of shoots with 5 µM *m*T allowed to obtain the highest content of rebaudioside A (10.74 mg/g DW). The lowest content of reabudioside A was observed in shoots cultured in the medium with the addition of BAP (on average 4.33 mg/g DW) (Table 2). Much like the case of stevioside, the accumulation of rebaudioside A per biomass of one bioreactor was the most effective in the case of 5 µM *m*T and the least effective on the control medium (49.31 mg/g DW, 11.80 mg/g DW, respectively). Similarly, among the tested cytokinins, BAP also inhibited rebaudioside A biosynthesis (on average, 14.95 mg/g DW). In this case, an average of 250.3 shoots was obtained per bioreactor (Tables 1, 2).

Our study showed that the significantly highest ratio of the rebaudioside A/stevioside was obtained for the stevia shoots cultivated in the medium enriched with 15 µM of zeatin (1.12), 15 µM kinetin (1.09) and 15 µM of *m*T (1.04). Slightly lower amounts were recorded when the shoots were treated with 5 µM of *m*T (0.94), 10 µM zeatin (0.98) and 5 µM zeatin (0.99) (Table 2). Generally, the SG ratio was the highest for shoots growing in the medium with the addition of zeatin, followed by *m*T, kinetin and BAP (Table 2).

### Influence of cytokinins on the accumulation of phenolic compounds

**Figure 2:**
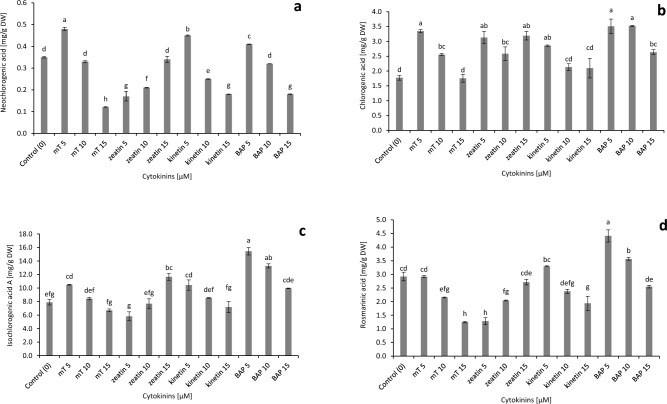
Effect of different cytokinins on phenolic acids biosynthesis in in vitro culture of *Stevia rebaudiana*: (**a**) neochlorogenic, (**b**) chlorogenic, (**c**) isochlorogenic A, (**d**) rosmarinic. Different letters indicate a significance difference at *P* < 0.05 according to ANOVA and Duncan’s test; the values are expressed as the means of three replicates (n = 3) ± standard deviation (SD); *m*T *meta*-Topolin, *BAP* 6-benzylaminopurine, *DW* dry weight.

The presence of phenolic acids—neochlorogenic, chlorogenic, isochlorogenic A and rosmarinic—and flavonoids—isoquercetin and quercitrin—was confirmed in all extracts from the in vitro stevia shoots treated with cytokinins and in the control shoots (Fig. 2, see Supplementary Fig. S2 online).

We observed that isochlorogenic acid A dominated (on average content: 9.50 mg/g DW), and neochlorogenic acid accumulated in the lowest amounts (on average content: 0.88 mg/g DW) in in vitro stevia shoots (Fig. 2).

As for the influence of cytokinins on the accumulation of phenolic acids, the highest content of chlorogenic acid was recorded in the case of shoots growing on the medium with the addition of 5 µM of BAP, 5 µM of *m*T and 10 µM of BAP (from 3.35 to 3.52 mg/g DW). A slightly lower value was noted in the case of shoots propagated on a medium enriched with kinetin (5 µM) and zeatin (5 and 15 µM) (on average, 3.06 mg/g DW). The lowest content was observed in the shoots treated with 15 µM of *m*T and in the control (1.76 and 1.78 mg/g DW, respectively). At a concentration of 5 µM, *m*T stimulated biosynthesis of neochlorogenic acid, while, at the highest concentration, it inhibited this process (content: 0.48 and 0.12 mg/g DW, respectively). For isochlorogenic acid A, the highest content was noted in shoots deriving from the medium with additions of 5 µM BAP (15.44 mg/g DW). Slightly less of this acid was obtained with 15 µM BAP (13.27 mg/g DW), and the lowest amounts were in the shoots treated with 5 µM zeatin (5.81 mg/g DW). A similar trend was observed in the case of rosmarinic acid, where 5 µM BAP stimulated (4.41 mg/g DW) and 5 µM zeatin inhibited (1.28 mg/g DW) biosynthesis. The addition of 15 µM of *m*T also had a negative impact (1.25 mg/g DW rosmarinic acid) (Fig. 2).

**Figure 3:**
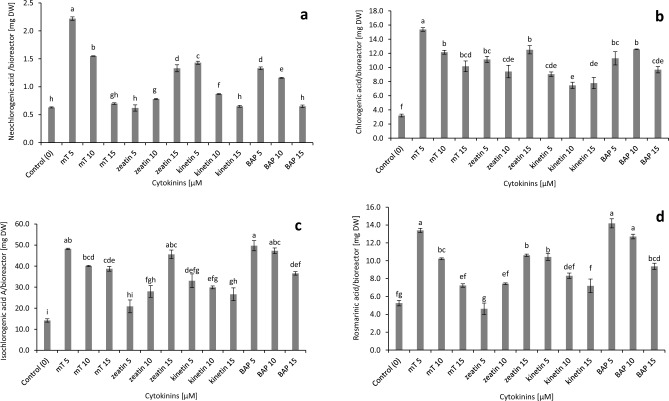
Effect of different cytokinins on phenolic acids accumulation in the bioreactor biomass of *Stevia rebaudiana*: (**a**) neochlorogenic, (**b**) chlorogenic, (**c**) isochlorogenic A, (**d**) rosmarinic. Different letters indicate a significance difference at *P* < 0.05 according to ANOVA and Duncan’s test; the values are expressed as the means of three replicates (n = 3) ± standard deviation (SD); *m*T *meta*-Topolin, *BAP* 6-benzylaminopurine, *DW* dry weight.

In the stevia culture, the accumulation of chlorogenic acid in the bioreactor DW biomass (4.59% DW, 351.50 shoots), as achieved with 5 µM of *m*T, was 4.8 times greater than that obtained in the control bioreactor biomass (1.80% DW, 151.50 shoots), neochlorogenic 3.52 times higher than control and rosmarinic acid 2.56 times higher than control. Here, 3.5 times more isochlorogenic acid A and 2.84 times more rosmarinic acid were reached from the total shoot biomass treated during four weeks with 5 µM BAP (3.22% DW, 245 shoots) compared with the control (Fig. 3, Table 1).

**Figure 4:**
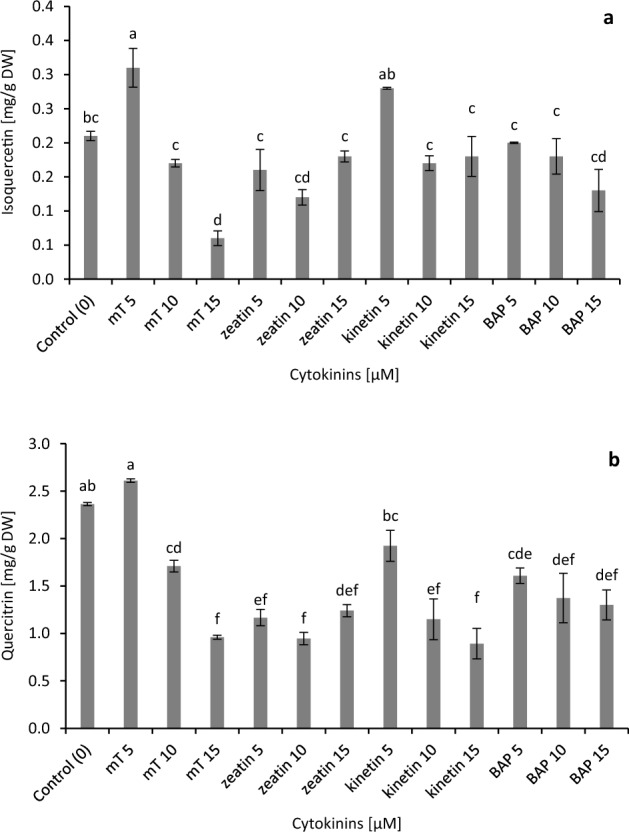
Effect of different cytokinins on flavonoids biosynthesis in in vitro culture of *Stevia rebaudiana*: (**a**) isoquercetin and (**b**) quercitrin. Different letters indicate a significance difference at *P* < 0.05 according to ANOVA and Duncan’s test; the values are expressed as the means of three replicates (n = 3) ± standard deviation (SD); *m*T *meta*-Topolin, *BAP* 6-benzylaminopurine, *DW* dry weight.

The concentration and type of cytokinins significantly influenced the production of individual flavonoids in the bioreactor culture of stevia (Fig. 4). The maximum contents of isoquercetin and quercitrin were obtained in medium supplemented with 5 µM of* m*T (0.31, 2.61 mg/g DW, respectively). A high level of isoquercetin was also observed under the influence of 5 μM kinetin and in the case of quercitrin in the control medium (0.28, 2.36 mg/g DW, respectively) (Fig. 4). The highest accumulation of flavonoids from one bioreactor biomass was identified in shoots after four weeks of culture in a medium enriched with 5 µM of *m*T.

**Figure 5:**
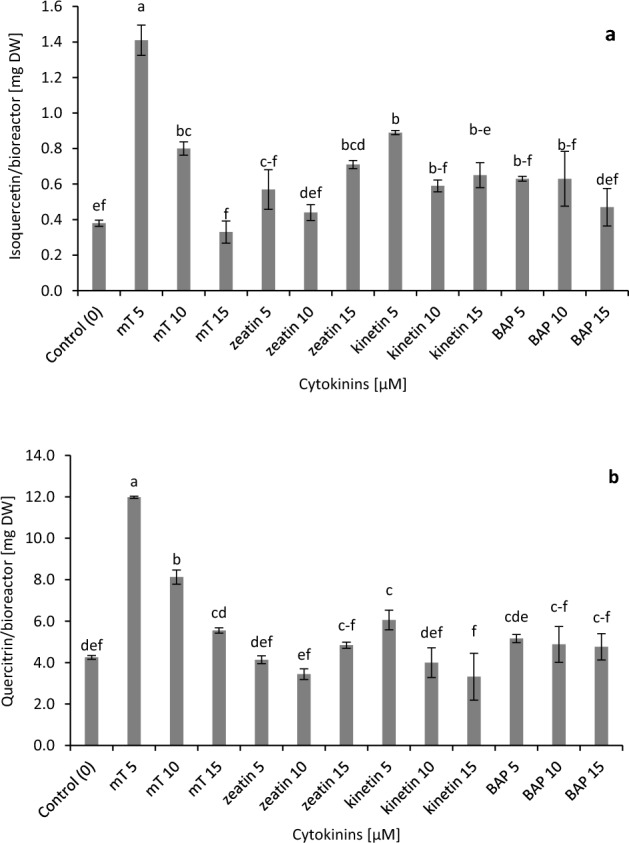
Effect of different cytokinins on flavonoids accumulation in the bioreactor biomass of *Stevia rebaudiana*: (**a**) isoquercetin and (**b**) quercitrin. Different letters indicate a significance difference at *P* < 0.05 according to ANOVA and Duncan’s test; the values are expressed as the means of three replicates (n = 3) ± standard deviation (SD); *m*T *meta*-Topolin, *BAP* 6-benzylaminopurine; *DW* dry weight.

The largest amounts of flavonoids from one bioreactor biomass were accumulated in shoots after four weeks of culture on a medium enriched with 5 µM *m*T. It is worth adding that, in this case, the production of isoquercetin was 3.71 times and quercitrin 2.82 times higher than in the control shoots (Fig. 5).

## Discussion

In the current study, the effect of the cytokinins: *m*T, zeatin, kinetin, BAP at concentrations of 0 (control), 5, 10 or 15 µM on *Stevia rebaudiana* Bertoni shoot appearance and multiplication, biosynthesis of SG and phenolic compounds in the temporary immersion bioreactor Rita^®^ were evaluated for the first time. The choices of cytokinin type and their concentrations were based on our preliminary studies with stevia and *Leucojum aestivum* cultures^28^.

As plant growth regulators, cytokinins have the ability to stimulate cell division and cause the development of shoots from the axillary buds. They also increase photosynthetically active pigments and are effective in delaying chlorophyll degradation^35^. Although it is known that shoot multiplication can also be regulated by endogenous cytokinins^36^, in the case of stevia, the presence of exogenous cytokinin in the medium seems to be necessary. Stevia shoots grown in a medium without the addition of cytokinins were necrotic and tended to turn brown and die. It is also worth emphasising that the stevia shoots growing in the medium containing the tested cytokinins did not show the symptoms of vitrification. So far, studies on the effect of BAP, kinetin and thidiazuron in stevia cultures have been carried out only on solid media^27,37^. To solve the problem of vitrification, which often occurs in cultures grown in a liquid medium, especially those enriched in cytokinins, temporary immersion systems like bioreactor Rita^®^ have been proposed in many studies^38^. Vilariño et al.^39^ found that a temporary immersion system can be applied for micropropagation of stevia. However, it was necessary to check the effect of various cytokinins used for the propagation of stevia in this type of bioreactor.

In the present study, a significant impact of the cytokinin on shoot multiplication was observed. The highest number of shoots was obtained when 5 µM of *m*T was added to the medium. Slightly fewer shoots were recorded using the medium containing 10 and 15 µM *m*T. *M*T has not yet been tested in in vitro cultures of stevia. However, in recent studies, *m*T has been applied very frequently as an alternative to conventional cytokinins for the multiplications of shoots: *Salvia sclarea*^40^, *Allamanda cathartica*^29^, *Oxystelma esculentum*^31^, *Rheum* sp. ‘Malinowy’^41^, *Crinum malabaricum*^30^, *Crinum brachynema*^42^ and *Caralluma umbellate*^44^. Moreover, research on *Crinum brachynema*^42^ and *Aloe polyphylla*^45^, similar to the presented results, showed that the 5 µM of *m*T is more favourable for the multiplication of shoots than higher concentrations. In our experiment, the addition of kinetin to the liquid medium was the least effective for stevia axillary shoot induction. Singh and Dwivedi^37^ confirmed the weaker effect of kinetin compared with thidiazuron on the multiplication of stevia shoots on a solid medium.

Among the tested cytokinins, the highest increases in fresh biomass (on average, 63.03 g) and the highest accumulation of DW (on average 5.04%) of shoots were observed when adding *m*T. In *Pterocarpus marsupium* plantlets, FW and DW biomass increments were higher in *m*T-derived plants than in BAP-derived plants, which is consistent with our findings^46^. In turn, in in vitro cultures of *Aloe arborescens* and *Huernia hystrix,* the highest shoot FW was recorded in the treatment with BAP than with *m*T^47,48^. The probable difference in* m*T action was because of the genotype difference. However, BAP can cause abnormal growth and hyperhydricity in shoots^49^. Of the tested concentrations of *m*T, 15 µM affects the highest increments of their FW and accumulation dry biomass. This may be because, under the influence of 15 μM *m*T, we also observed callus formation on the base of shoots, which could have affected the total biomass increments, especially because the number of shoots obtained in this case was fewer than when 5 μM of *m*T was used (21.20 and 23.43 shoots per explant, respectively). It is also worth emphasising that the accumulation of dry biomass results maintained the same pattern as those for the FW for all combinations used, proving that, by using the Rita^®^ bioreactor, we completely avoided the problem of hyperhydricity.

Stevioside and rebaudioside A are the best known and the most abundant SG in stevia^50^. It has been reported that, in natural conditions, the biosynthesis of SG primarily occurs in leaves and that they are transported to other plant organs^51^. The stevioside content varies from 5 to 22% while rebaudioside A from 22 to 61.6%, depending on the genotype and cultivation conditions^50^. Sharma et al.^52^ presented detailed information on the possibility of the biosynthesis of SG also by in vitro culture of stevia. Many factors, especially abiotic and biotic elicitors, affect the content of these metabolites in in vitro condition^24^. However, there is little information about the effect of plant growth regulators, including cytokinins, on SG biosynthesis in stevia shoots grown in liquid media. Röck-Okuyucu et al.^26^ showed that a solid medium without growth regulators led to considerably higher stevioside content compared with the plant growth regulators (BAP, kinetin, thidiazuron, IAA (Indole-3-acetic acid) and NAA (α -naphthalene acetic acid)) containing media. While rebaudioside A was only present in plant material derived from medium without growth regulators. Pazuki et al.^27^ noted no differences in the content of stevioside and rebaudioside A in shoots derived from a solid medium enriched with kinetin and BAP compared with a medium without cytokinins. On the other hand, cytokinins may stimulate the biosynthesis of secondary metabolites. The effect of cytokinins may also be different in the case of cultures grown on solid and liquid media^28^.

To increase the biosynthesis of SG, *m*T—as an alternative to conventional cytokinins—was used for the first time. The data obtained in our study showed that 5 µM of *m*T stimulates the biosynthesis of both stevioside and rebaudioside A. *M*T is not yet widely used for the production of metabolites. For example, *m*T increased the levels of chlorophyll, soluble proteins and free amino acids in shoots of *Daphne mezereum*^53^. Additionally, *m*T stimulates galanthamine biosynthesis in *Leucojum aestivum* plants cultivated on solid medium^28^.

Although the production of secondary metabolites in in vitro cultures usually takes place in two stages, the first stage is biomass accumulation, and the second stage concerns the production of secondary metabolites. In the presented studies, 5 µM *m*T stimulated both shoots multiplication, DW biomass content, and SG biosynthesis. On the other hand, in the medium without the addition of cytokinins, we obtained shoots characterised by a high content of SG and the fewest shoots and biomass increments. This also confirms the observation that unfavourable conditions for plant growth and development cause stress reactions, thus stimulating the biosynthesis of secondary metabolites^54^. In the presented studies, we observed that the addition of BAP to the medium had a negative effect on the content of rebaudioside A obtained in biomass from one bioreactor, though it is worth emphasising, however, that while a large number of shoots was obtained (only less than in the case of *m*T treatment), their combined weight was low.

The presence of 15 µM of zeatin, kinetin and *m*T led to the highest ratio of rebaudioside A/stevioside (on average 1.08). The effect of cytokinins on the stevioside ratio in shoot cultures has not yet been determined. Blinstrubienė et al.^22^ showed that in stevia callus cultures the rebaudioside A-to-stevioside ratio ranges from 0.03 to 1.0. According to the literature, for stevia growing in natural conditions, the rebaudioside A-to-stevioside ratio is 0.37–0.86^55^. Because rebaudioside A has better sweetening properties and quality of taste than stevioside, the rebaudioside A-to-stevioside ratio should be as high as possible^56^.

In our study, in stevia shoot biomass extracts, four phenolic acids—neochlorogenic, chlorogenic, isochlorogenic A (3,5-dicaffeoylquinic acid) and rosmarinic—and two flavonoids—isoquercetin and quercitrin—were determined. It is worth noting that the research on the biosynthesis of individual phenolic compounds in in vitro cultures of stevia is incomplete. Fu et al.^19^ showed that chlorogenic acid, 3,5-dicaffeoylquinic acid (isochlorogenic acid A) and 4,5-dicaffeoylquinic acid were produced by the hairy roots of stevia. As for the content of phenolic acids in stevia leaves, which are derived from plants growing in natural conditions, more than 30 of them have been isolated^20^; among them, chlorogenic, caffeic and trans-ferulic acids are the main phenolic acids present in fresh and dried stevia leaves^57^. Isochlorogenic acid A was found to be dominant in the investigated shoot cultures, whereas neochlorogenic acid was found to accumulate in the lowest amounts. As in the present research, Simlat et al.^34^ also noted that, in stevia plants obtained from seeds, among all analysed phenolic acids, isochlorogenic A were present in the largest amounts. Isochlorogenic acid A has been identified in various species and currently shows great interest because of its medical properties: antiviral, neuroprotective, antioxidant, hepatoprotective and antihepatitis B. The protective effect of isochlorogenic acid A on liver fibrosis has also been observed^58,59^. The results of the RP-HPLC analyses indicate that 5 µM of BAP was a good biosynthetic elicitor for chlorogenic, isochlorogenic A and rosmarinic acids, whereas 5 µM of *m*T was effective for chlorogenic and neochlorogenic acids. BAP is a cytokinin used to stimulate the biosynthesis of rosmarinic acid and salvianolic acid in in vitro culture of *Dracocephalum forrestii*^60^. While *m*T, applied at lower concentrations (2.5 and 5 µM) similar to the present study, has been shown to contribute to an increase in the content of phenolic acids (chlorogenic acid, ferulic acid, *p*-coumaric acid and sinapic acid) in in vitro cultures of *Crinum malabaricum*^30^.

Among the flavonoids found in stevia leaves, for example, are hispidulin, 6-methoxyluteolin, quercetin, avicularin, rutin, hyperoside, luteolin, quercetin-3-D-glycoside, kaempferol, apigenin and quercitrin^34^. However, in in vitro cultures of stevia, only the total content of flavonoids was determined^22,23^.

Our results demonstrate that 5 µM *m*T stimulated the production of isoquercetin and quercitrin in bioreactor shoot cultures of stevia. The present paper is, to the best of our knowledge, the first report describing the effect of cytokinin, including *m*T, on individual flavonoid biosynthesis in stevia cultured in bioreactor Rita. There is also little data on the influence of *m*T on the content of individual flavonoids in other species. The positive effect of *m*T on the total flavonoid production was noted for example in shoot cultures of *Allamanda cathartica*^29^.

## Conclusion

Our research, which was carried out in bioreactor Rita^®^, shows the positive effect of 5 µM *m*T on *Stevia rebaudiana* shoot multiplication and production of stevioside, rebaudioside A, chlorogenic and neochlorogenic acids and flavonoids. Here, 5 µM BAP stimulated biosynthesis chlorogenic, isochlorogenic A and rosmarinic acids. However, the amount of these phenolic acids accumulated in biomass from one bioreactor obtained after four weeks of culture was the same or lower under the presence of 5 µM BAP as when 5 µM *m*T were used in the medium. The results clearly indicate for the first time that *m*T is the best cytokinin of all those tested in the in vitro culture of stevia. To optimise the mass production of stevia plants and secondary metabolites, different *m*T application times in bioreactor culture will have to be investigated in detail in future studies.

### Supplementary Information

Supplementary Figures.

## Data Availability

The datasets generated during the current study are available from the corresponding author on reasonable request.
