# Supplementary material for: Meta-Topolin-induced mass shoot multiplication and biosynthesis of valuable secondary metabolites in Stevia rebaudiana Bertoni bioreactor culture
Source: Sci Rep. 2023 Sep 19;13:15520. doi: 10.1038/s41598-023-42619-8 (PMC10509197; doi:10.1038/s41598-023-42619-8)
Supplement: Supplementary file 1 — Supplementary Figures. [file 41598_2023_42619_MOESM1_ESM.pdf]

Supplementary materials for:

Meta-Topolin–induced mass shoot multiplication and biosynthesis of valuable secondary metabolites in *Stevia rebaudiana* Bertoni bioreactor culture

Agata Ptak[1*], Agnieszka Szewczyk[2], Magdalena Simlat[1], Alicja Błażejczak[1] & Marzena Warchoł[3]

[1]Department of Plant Breeding, Physiology and Seed Science, University of Agriculture in Krakow, Łobzowska 24, 31-140 Krakow, Poland
[2]Department of Pharmaceutical Botany, Faculty of Pharmacy, Jagiellonian University Medical College, Medyczna 9, 30-688 Krakow, Poland
[3]The Franciszek Górski Institute of Plant Physiology, Polish Academy of Sciences, Niezapominajek 21, 30-239 Krakow, Poland

*Correspondence: mfptak@cyf-kr.edu.pl, agata.ptak@urk.edu.pl (A. Ptak)

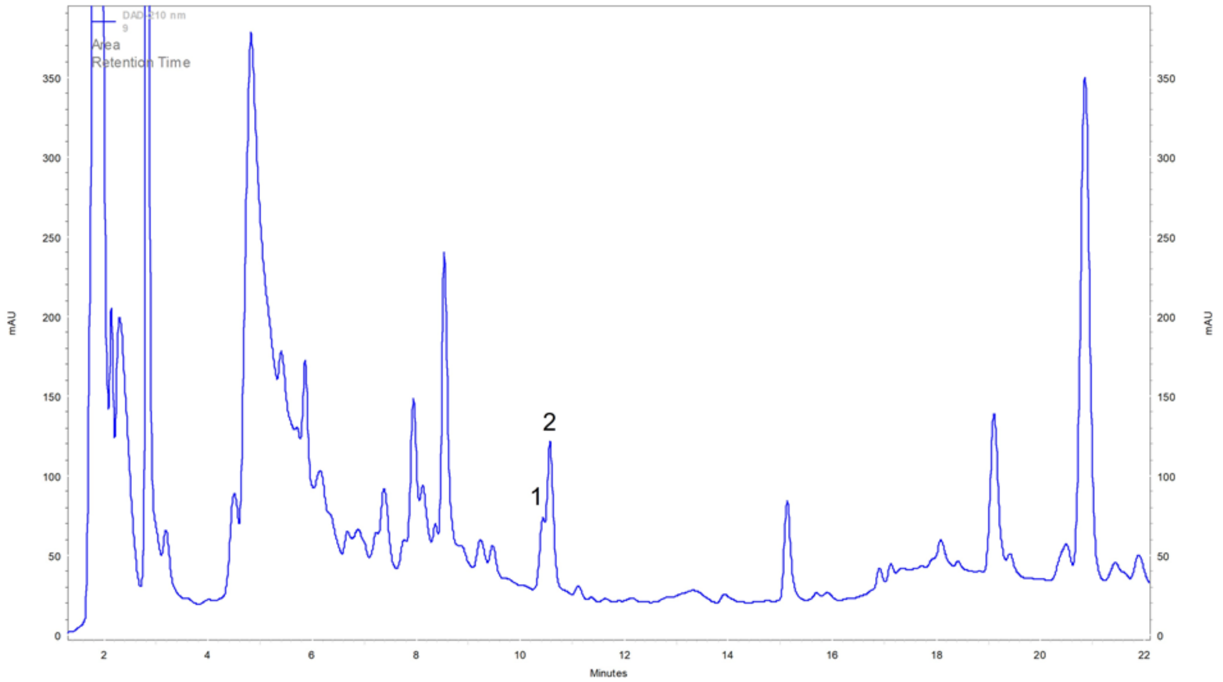

Figure S1. HPLC chromatogram (analysis of steviol glycosides) of methanolic extract of *Stevia rebaudiana* (sample: in vitro shoots from 5 µM of *meta*-Topolin) 1. rebaudioside A, 2. stevioside.

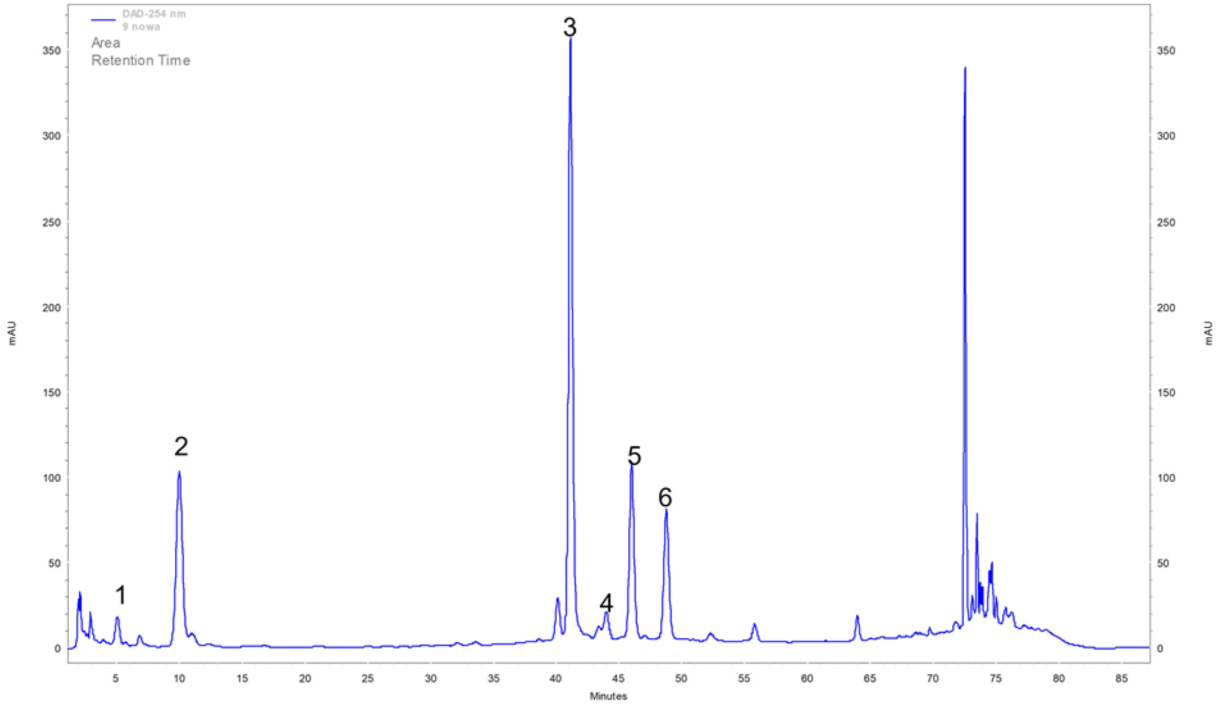

Figure S2. HPLC chromatogram (analysis of phenolic compounds) of methanolic extract of *Stevia rebaudiana* (sample: in vitro shoots from 5 µM of *meta*-Topolin); 1. neochlorogenic acid, 2. chlorogenic acid, 3. isochlorogenic acid A, 4. isoquercetin, 5. rosmarinic acid, 6. quercitrin.
